# Mechanistic PKPD modeling to describe cytokine release associated with CD3 T-cell engager therapies

**DOI:** 10.3389/fimmu.2024.1463915

**Published:** 2025-01-17

**Authors:** Apolline Lefèvre, Zinnia P. Parra-Guillen, Iñaki F. Trocóniz, Christophe Boetsch, Nicolas Frances

**Affiliations:** ^1^ Roche Pharma Research and Early Development (pRED), Pharmaceutical Sciences PS, Roche Innovation Center Basel, Basel, Switzerland; ^2^ Pharmacometrics & Systems Pharmacology, Department of Pharmaceutical Science, School of Pharmacy and Nutrition, University of Navarra, Pamplona, Spain; ^3^ Navarra Institute for Health Research (IdiSNA), Pamplona, Spain; ^4^ Institute of Data Science and Artificial Intelligence (DATAI), University of Navarra, Pamplona, Spain

**Keywords:** cancer immunotherapy (CIT), T-cell engager (TCE), cytokine release, pharmacokinetics/pharmacodynamics (PK/PD) modeling, effector T-cell dynamics

## Abstract

**Introduction:**

T-cell engagers (TCE), a therapeutic class of cancer immunotherapy (CIT), offer a novel approach to cancer treatment by harnessing and reactivating the patient’s immune system to eradicate tumor cells. However, the use of TCE in the clinic can lead to severe side effects, including cytokine release syndrome (CRS). Therefore, innovative dosing strategies need to be implemented to mitigate the risk of developing CRS.

**Method:**

In the presented work, a mechanistic pharmacokinetics/pharmacodynamics (PKPD) model describing cytokine release following TCE therapy has been developed combining literature knowledge and preclinical data. The model was developed to explore and test hypotheses regarding the mechanisms behind the decrease of cytokine release following two repeated TCE administrations.

**Results:**

The model is able to successfully reproduce the observed dynamics of cytokine levels associated with the initial and subsequent TCE doses, accounting for different dosing intervals. In addition, the model suggests a mechanism of action that uncouples cytokine release from tumor cell killing.

**Discussion:**

This model provides an initial mechanistic framework to support the design of experiments and paves the way for the application of mathematical modeling to support clinical dosing regimen selection of any TCE.

## Introduction

Cancer immunotherapy (CIT) has recently drawn remarkable attention by showing its ability to improve the overall survival in the clinic (1) and has led to the approval of more than 40 drugs by health authorities (2). Various classes of therapeutic agents exist in this area and share the concept of enhancing the existing anti-tumor immunity to eradicate malignant cells (3). One approach is to use synthetic immunity by redirecting the T-cells against the tumor cells (1). T-cell engagers (TCE) are bispecific antibodies (bsAbs) (4) that bind simultaneously to T-cells via the CD3 epsilon chain (CD3) within the T-cell Receptor (TCR) complex and to tumor cells via tumor-associated antigen (TAA) (5). The crosslinking of a tumor cell with a T lymphocyte enables the formation of an immunological synapse, similar to that of a natural TCR and peptide–major histocompatibility complex (MHC) complex (6). The synapse formation redirects any engaged T-cells in an inflammatory process (cytokine release, triggering further immune activity) and tumor killing.

TCEs are thus promising anti-tumor therapies but have remaining challenges associated with their clinical safety profiles. The primary toxicities following their administration are immune effector cell-associated neurotoxicity syndrome (ICANS) and cytokine release syndrome (CRS) (7). The latter is generally associated with cytokine release upon T-cell activation (8). Patients experiencing CRS may have a variety of symptoms (9) for which the grading has been defined by the Common Terminology Criteria for Adverse Events (CTC-AE) v5 (10). CRS incidence (grade I or more) in clinical trials of FDA approved bispecific TCEs ranged from 15% to 89% (11).

Strategies are implemented in the clinic to mitigate CRS, such as step-up dosing (SUD) (12), which involves gradually increasing the dose within the same patient until reaching the target dose; the use of a cytokine blockage agent (e.g. Tocilizumab (13), an anti-IL6 antibody) (14) or the use of corticosteroids (e.g. Dexamethasone) (15). In addition, *in vitro* data suggest that SUD can be used as a prophylactic method to prevent CRS in the clinical setting (16). Selection of the best dosing regimen, particularly in the context of SUD, is currently based on empirical methods. Mathematical models, including the proposed mechanistic PKPD model, can be used to inform dosing regimen selection and have the potential to reduce both the length of dose escalation trials and the empirical explorations of dose and schedule.

Some mathematical models predicting cytokine release after TCE administration have been previously published (17–19). Those models describe the decrease in cytokine release observed upon repeated TCE administration as a consequence of previous target cell depletion. The model from Chen and colleagues (19) additionally assumes that cytokines, once released, inhibit the release of new cytokines via a negative feedback loop. The hypothesis that the loss of cytokine release is a consequence of the T-cell killing of target cells was refuted by the work of Li et al. (16). They demonstrated *in vitro* that, when a second TCE stimulation was done seven days after the first one, lower levels of cytokines were associated with the second dose versus the first one while cytotoxic activity was conserved. This was observed despite the *in vitro* system being re-supplemented with fresh tumor cells prior to the second stimulation. Alternatively, the pool model proposed by Movin-Osswald and Hammarlund-Udenaes (20) could provide another hypothesis for the reduction in cytokine release following two TCE administrations. The original pool model assumes the existence of an initial reservoir (a pool) of available prolactin (PRL) that is depleted upon remoxipride administration, thus preventing subsequent releases upon multiple dosing until the pool has been replenished. Although this approach has not been previously applied within the context of CRS, it could be adapted by considering the cytokines as a similar pool.

Hereby, a hypothesis-driven mechanistic PKPD model is presented with the aim of exploring potential mechanistic insights behind cytokine release upon the two first TCE administrations. The proposed PKPD model expands upon previously published models (17–20) by incorporating evidence from newly available published preclinical data (16). The presented model focuses on predicting cytokine release upon the first two consecutive TCE administrations with varying dosing intervals and uncouples cytokine levels from tumor burden.

## Materials and methods

### Preclinical literature data

The PKPD model was developed based on literature preclinical data from Li et al. (16) where mechanisms of cytokine release were investigated. The group performed an *in vivo* study in which mammary tumor-bearing mice were treated with two subsequent doses of anti-HER2 T-cell Bispecific (0.5mg/kg) at different dosing intervals (administrations at day 0 and day 1; day 0 and day 7; day 0 and day 14; day 0 and day 21 or day 0 and day 28). Systemic cytokine release (interleukin-6, IL6) was monitored two hours after each treatment. Published data from Li et al. (16) were digitized using WebPlotDigitizer (21) and are available in the appendix section (**Supplementary Table S1**).

### PKPD model development

**Figure 1:**
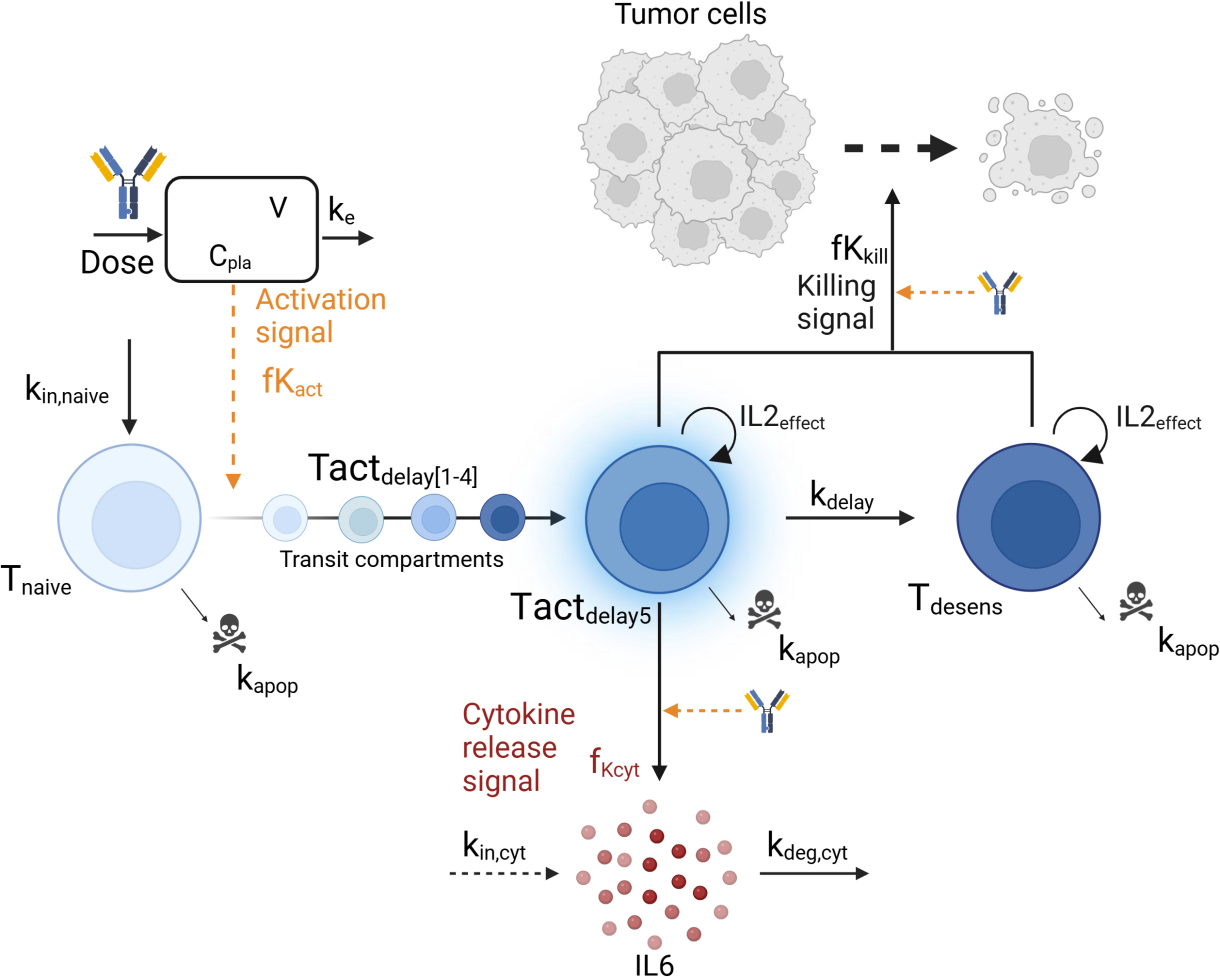
Graphical representation of the mechanistic pharmacokinetic/pharmacodynamic (PK/PD) model. The model describes the T-cell engager (TCE) plasma concentration (C_pla_) and its impact on the transition from naive T-cells (T_naive_) to activated T-cells (T_act_), leading to cytokine release (IL6) and tumor cell killing. Biological entities and parameters are described in the text.

A mechanistic PKPD model was built using a bottom-up approach including the main biological players involved in cytokine release: TCE, tumor cells, T-cells and interleukins. The model (**Figure 1**) assumed that upon TCE exposure, T-cells are activated and transition through different states in which they can trigger cytokine release and kill tumor cells, as supported by (22). The dynamics of the different entities were modeled using ordinary differential equations (ODE) describing the mass balance between the synthesis, transition and degradation or apoptosis processes and implemented using zero-, first- or second-order rate constants. Michaelis-Menten or Hill kinetic functions were also implemented to account for saturation processes. Given the limited available pre-clinical data to build the model, parameters could not be estimated. Model parameters characterizing the above mentioned processes were taken from literature or fine tuned (calibrated) to describe the available data of *in vivo* IL6 (16). Some parameters had to be borrowed from human references to simulate the PKPD model in mice.

### Model evaluation and exploration

Model performance was graphically assessed by confronting model predictions to the *in vivo* digitized data from (16). The unique IL6 sample (two hours following TCE administration) was assumed to be reflective of the maximum IL6 levels (23). In addition, the cytokine release and killing functions were explored under different relevant dosing scenarios to understand the behavior of the system (same dose levels, 0.5mg/kg, and different dosing intervals). Predictions from the PKPD model were also compared to simulations derived from a simpler model, the pool model from (20), which was repurposed to describe cytokine release (Appendix). Lastly, a parameter sensitivity analysis was performed to evaluate the effects of varying each parameter individually (+/- 20%) (24) on the ratio of maximum cytokine release at second TCE administration versus first administration, and identify the most influential parameters. Parameters involved in tumor cell killing were not included in the sensitivity analysis as they have no impact on cytokine release. Model simulations (ODE computations) were conducted in Rstudio version 4.1.3 (25) using the RxODE2 package (26).

## Results

### Model description

The structure of the developed mechanistic PKPD model is presented in **Figure 1**.

#### Pharmacokinetics

The TCE plasma concentration (C_pla_) was assumed to follow a 1-compartment PK model characterized by a linear clearance (CL) and distribution volume (V) parameters from a similar anti-HER2 TCE (27) (Equation 1). Initial drug concentration prior to dosing, C_pla_(t=0), is equal to zero.


(1) $$\frac{dC_{pla}}{dt}=\;-\frac{CL}{V}\times C_{pla}$$

$$C_{pla}\left(t=0\right)=0$$

#### T-cell dynamics

Three T-cell states were included in the model: baseline naive T-cells (T_naive_), activated T-cells (T_act_) and desensitized T-cells (T_desens_). Baseline naive T-cells (Equation 2) are activated upon TCE exposure as follow:


(2) $$\frac{dT_{naive}}{dt}=k_{in,naive\;}-\left(k_{apop}+fK_{act}\right)\times T_{naive}$$

$$T_{naive}\left(t=0\right)=\;\frac{k_{in,naive\;}}{k_{apop}}$$

where k_in,naive_ and k_apop_ are the zero and first-order rate constants for baseline naive T-cells input and apoptosis, respectively. At baseline, the amount of naive T-cells T_naive_(t=0) is given by k_in,naive_/k_apop._ The activation signal, fK_act_, described in Equation 3 follows an E_max_ -like relationship with respect to the TCE plasma concentration, characterized by a Hill coefficient h_act_, a maximum activation signal E_max,act_ and an EC50_act_, which is the TCE concentration required to reach half of maximal T-cell activation. As only one dose level (0.5mg/kg) was tested *in vivo*, the model assumes a complete transfer of the naive T-cells to activated T-cells at the tested dose. The parameter values should be adjusted with more evidence from new experiments.


(3) $$fK_{act}=E_{max,act}\times \left(\frac{C_{pla}^{h_{act}}}{C_{pla}^{h_{act}}+EC50_{act}^{h_{act}}}\right)\;$$

The transition from baseline naive T-cells to activated T-cells state (Tact_delay_) was modeled with 5 transit compartments (Tact_delay1_ to Tact_delay5_) to account for the lag time between maximum TCE exposure and maximum cytokine level, and is governed by Equations 4, 5. At baseline, no activated T-cells are present and Tact_delay_(t=0) = 0. The parameter value for the first-order transit rate constant k_delay_ between the transit compartments was selected to mimic a delay of a few hours between drug administration and cytokine release (16). Activated T-cells (Tact) undergo apoptosis at the same rate parameter as naive T-cells, k_apop_ (see below).


(4) $$\begin{array}{c} \frac{dTact_{delay1}}{dt}=fKact\times T_{naive}-k_{delay}\times Tact_{delay1}-k_{apop}\\ \times \;Tact_{delay1}+IL2_{effect}\times Tact_{delay1} \end{array}$$

(5) $$\begin{array}{c} \frac{dTact_{delay5}}{dt}=k_{delay}\times (Tact_{delay4}-\;Tact_{delay5})-k_{apop}\\ \times \;Tact_{delay5}+IL2_{effect}\times Tact_{delay5} \end{array}$$

$$\begin{array}{c} Tact_{delay1}\left(t=0\right)=\;Tact_{delay2}\left(t=0\right)\;=\;Tact_{delay3}\left(t=0\right)\\ =\;Tact_{delay4}\left(t=0\right)=\;Tact_{delay5}\left(t=0\right)=0 \end{array}$$

The proliferation of T-cells was assumed to be associated with an IL2 cytokine release upon TCE activation, as IL2 secreted by T-cells binds to T-cell receptors and induces their clonal proliferation (28). An IL2_effect_ variable was therefore introduced (Equations 4, 5) mimicking an autocrine loop for T-cell proliferation in presence of IL2 as follow:


(6) $$IL2_{effect}=k_{IL2}\times \left(1-I_{max}\times \frac{{\left(E:T\right)}^{h_{IL2}}}{{\left(E:T\right)}^{h_{IL2}}+EC50_{E:T,IL2}^{\;h_{IL2}}}\right)$$

where k_IL2_ is the maximum proliferation rate parameter of T-cells induced by IL2. In turn, this process can be inhibited when effector cells (T-cells) outnumber target cells (29). Hereby, T-cell proliferation by IL2 (Equation 6) is inhibited by a maximum inhibition rate parameter (I_max_), an EC50_E:T,IL2_ parameter which correspond to the Effector to Target (E:T) ratio parameter needed to achieve half of maximum inhibition IL2 effect and a shape parameter (h_IL2_). All T-cells (naive, activated and desensitized) as well as all tumor cells are accounted for in the E:T ratio. For this analysis and in absence of clinical information, the initial values of T-cells and tumor cells were selected to match an optimal *in vitro* E:T ratio of 10 (16).

#### Cytokine release

Only activated T-cells from the fifth T-cells activated transit compartment (Tact_delay5_) release cytokine (IL6), Cyt, (Equation 7) according to the following equation:


(7) $$\frac{dCyt}{dt}=k_{in,cyt}\times \left(1+fK_{Cyt}\right)-k_{deg,cyt}\times Cyt$$

Cyt(t=0)=10pg/mL

in which k_in,cyt_ and k_deg,cyt_ are zero and first-order rate constants representing the physiological production and degradation of cytokines, respectively. Baseline cytokine levels, Cyt(t=0), are set to 10pg/mL (30) and defined by k_in,cyt_/k_deg,cyt_. Function fK_Cyt_ (Equation 8) describes the increase of cytokine production upon T-cell activation as follow:


(8) $$fK_{Cyt}=E_{max,cyt}\times \left(\frac{C_{pla}^{\;h_{cyt}}}{C_{pla}^{\;h_{cyt}}+EC50_{cyt}^{\;h_{cyt}}}\right)\;\times Tact_{delay5}^{\;\alpha }$$

Where E_max,cyt_ is the maximum increase in cytokine production, h_cyt_ is the Hill coefficient representing the steepness of the exposure-cytokine release relationship, and EC50_cyt_ is the TCE concentration required to reach half of maximal cytokine production increase. In Equation 8, cytokine release also depends on the amount of Tact_delay5_ and the shape parameter α. Those parameters were calibrated to describe the *in vivo* data from Li et al. (16).

Subsequently, the activated T-cells transit into a desensitized T-cell status, T_desens_, which represent T-cells that retained their ability to kill tumor cells without releasing cytokines (Equation 9). This desensitization process might be driven by the release of cytokine itself. As an example, the release of IL10, a regulatory cytokine, is thought to inhibit the release of more pro-inflammatory cytokines like IL6 and TNFα (31). Baseline levels of desensitized T-cells, T_desens_(t=0), are set to zero. The same parameter value (k_delay_) has been assumed for the desensitization rate, in absence of data to differentiate them.


(9) $$\frac{dT_{desens}}{dt}=k_{delay}\times Tact_{delay5}-k_{apop}\times \;T_{desens}+IL2_{effect}\times T_{desens}$$

$$T_{desens}\left(t=0\right)=0$$

It has been hypothesized that desensitized T-cells are, as activated T-cells, affected by the T-cell proliferation effect (IL2_effect_) (Equation 6).

#### Tumor cell killing

Li et al. (16) demonstrated *in vitro* that reduced cytokine release upon the second dose was independent of tumor cell depletion and did not compromise cytotoxic efficacy. Tumor cell growth and killing were included in the model to determine if the observed *in vitro* data could be explained by the hereby proposed mechanisms. Tumor cells (Tumor) were assumed to follow an exponential growth (Equation 10) with a growth rate k_g_. They are killed by activated (Tact_delay1_, …) and desensitized (T_desens_) T-cells as determined by the fK_kill_ killing function (Equation 14) following an Emax relationship versus drug concentration (C_pla_, EC50_kill_) and on the number of T-cells available for killing (T_kill_, Equation 15). It was assumed that the TCE was necessary to modulate the activation of the T-cells and thus, no baseline killing was described when no drug was administered. Tumor cell killing was modeled with 3-transit compartments (Equations 11-13, Tumor1 to Tumor3) with a delay of k_delay,tumor_ (32).


(10) $$\frac{dTumor}{dt}=Tumor\times \left(k_{g}-fK_{kill}\right)$$

$$Tumor\left(t=0\right)=0.1*T_{naive}\left(t=0\right)$$

(11) $$\frac{dTumor1}{dt}=fK_{kill}\times Tumor-k_{delay,tumor}\times Tumor1$$

(12) $$\frac{dTumor2}{dt}=k_{delay,tumor}\times \left(Tumor1-Tumor2\right)$$

(13) $$\frac{dTumor3}{dt}=k_{delay,tumor}\times \left(Tumor2-Tumor3\right)$$

Tumor1(t=0)=Tumor2(t=0)=Tumor3(t=0)=0

(14) $$fK_{kill}=E_{max,kill}\;\times \;\left(\frac{C_{pla}}{C_{pla}+EC50_{kill}}\right)\times T_{kill}$$

(15) $$\begin{array}{c} T_{kill}=Tact_{delay1}+Tact_{delay2}+Tact_{delay3}+Tact_{delay4\;}\\ +Tact_{delay5}+T_{desens} \end{array}$$

As no tumor data was available from the *in vivo* experiments, growth rate (k_g_) and transit delay (k_delay,tumor_) were taken from the literature (32) while plausible values were selected for the E_max,kill_ and EC50_kill_.

The model parameters of the mechanistic PKPD model are displayed in **Table 1**.

**Table 1:** Mechanistic PKPD model parameters (in mince) and their corresponding values, units and source (literature or calibrated with the available *in vivo* data).

	Parameter	Value	Units	Source/method	Description
PK	V	2.3	mL	Adapted from Yu et al. (27)	Volume of the central compartment
	CL	0.025	mL/h		Elimination rate constant
T-cell kinetics	k_in,naive_	16	cells/uL/h	Lythe et al. (38) (theoretical computations)	Synthesis rate of naive T-cells *
	k_apop_	0.01	1/h	Adapted from Friberg et al. (39)	Apoptosis rate of naive T-cells *
T-cell activation	E_max,act_	1.5	1/h	Calibrated	Maximum of T-cell activation signal
	h_act_	2	–	Calibrated	Hill coefficient for activation signal
	EC50_act_	1	µg/mL	Calibrated	Exposure of TCE to achieve half of maximal T-cell activation
	k_delay_	2.5	1/h	Adapted from Li et al. (16), Friberg et al. (39)	Transit rate between compartments
Cytokine release	k_in,cyt_	10* k_deg,cyt_	1/h	Frances et al. (30)	Synthesis rate of cytokines **
	k_deg,cyt_	0.41	1/h		Degradation rate of cytokines **
	E_max,cyt_	0.00086	pg/mL/h	Calibrated	Maximum cytokine release from T-cells
	h_cyt_	2.5	–	Calibrated	Hill coefficient for cytokine release signal
	EC50_cyt_	5	µg/mL	Calibrated	Exposure of TCE to achieve half of maximal cytokine release from T-cells
	α	2	–	Calibrated	Exponent
T-cell proliferation by IL2	k_IL2_	0.0008	1/h	Macallan et al. (40)	Maximum proliferation rate of T-cells (activated and desensitized) induced by IL-2 *
	I_max_	1	–	Fixed	Maximum inhibition of IL2 effect
	h_IL2_	5	–	Fixed	Hill coefficient for IL2 effect
	EC50_E:T,IL2_	7	–	Adapted from Bacac et. al (5)	E:T to achieve half of maximal IL2 effect^a^
Tumor kinetics	k_g_	0.006	1/h	Simeoni et al. (32)	Tumor growth rate
	k_delay,tumor_	0.02	1/h		Transit rate between tumor compartments
	E_max,kill_	0.000025	1/cells/uL/h	Calibrated	Maximum killing of tumor cells
	EC50_kill_	1	µg/mL	Adapted from Staflin et al. (23)	TCE plasma concentration needed to achieve half of maximal tumor cell killing for the TCE concentration

a *In vitro* conditions for maximal cytotoxic effect, *From humans, **From cynomolgus monkeys.

### Model evaluation

**Figure 2:**
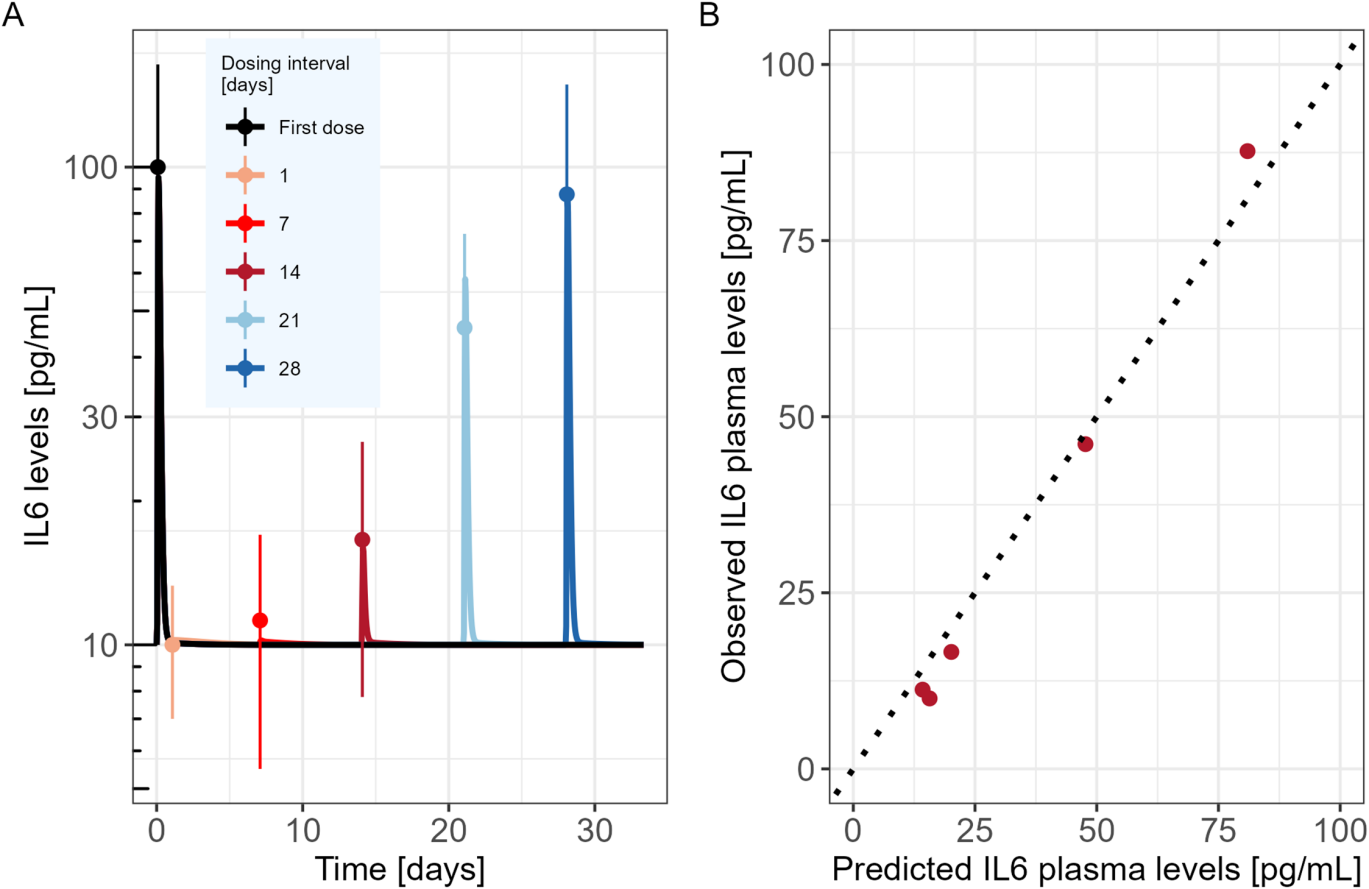
Mechanistic PKPD model performance **(A)** Model predicted IL6 time course in mice (solid lines) following two consecutive TCE administrations at different dosing intervals (administrations at day 0 and day 1; day 0 and day 7; day 0 and day 14; day 0 and day 21 or day 0 and day 28). Dots represent the digitized experimental data from Li et al. (2019) along with their standard error of mean (SEM). **(B)** Observed versus model predicted IL6 cytokine levels.

The developed model was used to simulate the time course of IL6 cytokine as in the experimental design of Li et al., i.e. following two consecutive TCE administrations given at different dosing intervals (administrations at day 0 and day 1; day 0 and day 7; day 0 and day 14; day 0 and day 21 or day 0 and day 28). Observed IL6 levels versus PKPD model predictions are shown in **Figure 2A**. The mechanistic PKPD model was capable of reproducing experimental data (16) with (1) a lower cytokine release after the second compared to the TCE first administration, (2) an increase in cytokine release at the 2nd TCE administration when increasing the dosing interval between the two administrations (3) and a similar cytokine levels between the first and second TCE administration when dosing interval is of 28 days. The observed versus predicted plot (**Figure 2B**) did not reveal any systematic bias. The model was also able to reproduce the dynamic of other cytokines such as TNFα (**Supplementary Figure S1**, Appendix), following repeated administration of TCE.

**Figure 3:**
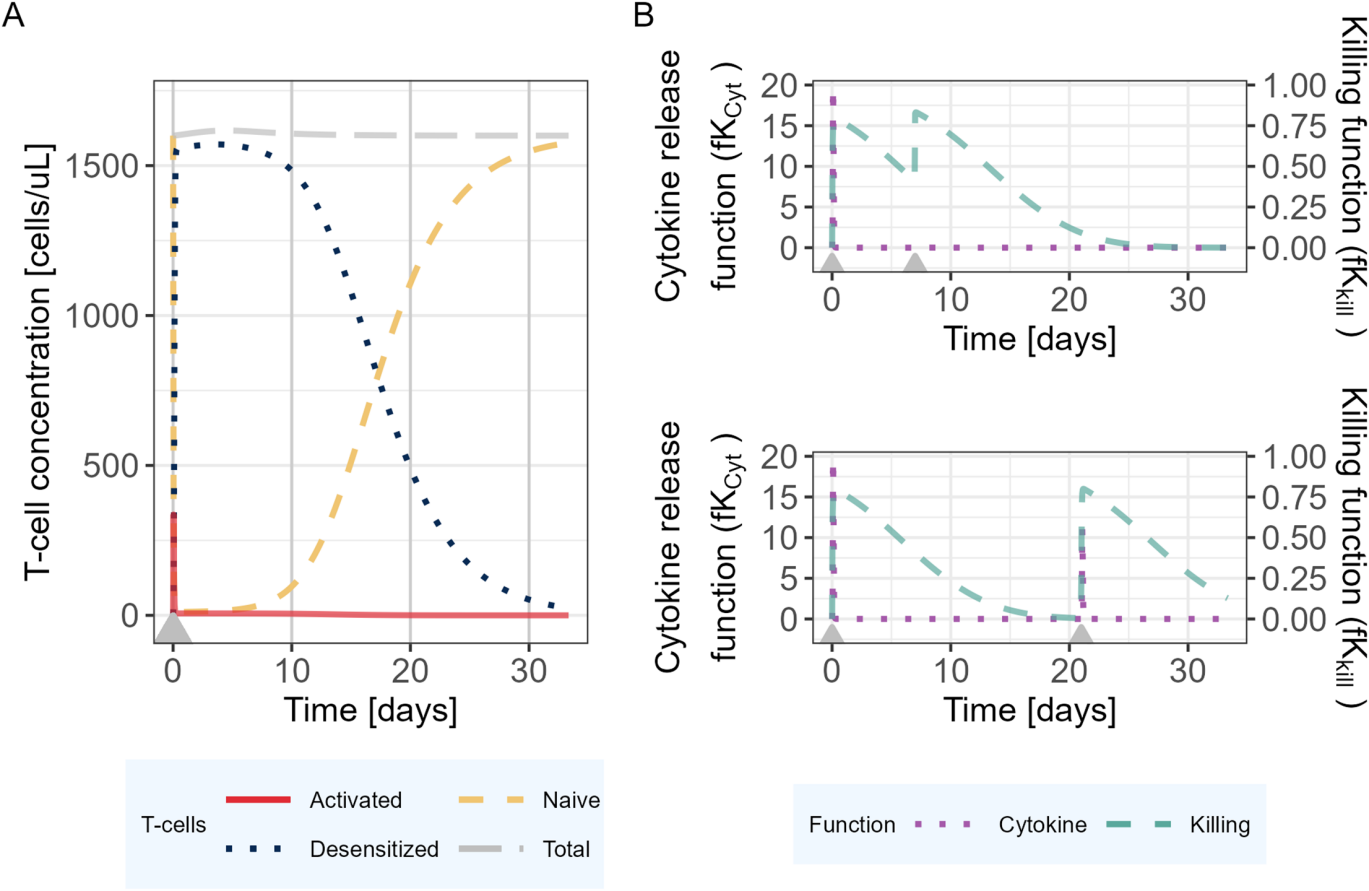
Mechanistic PKPD model simulations of **(A)** T-cell dynamics following one TCE administration on day 0. **(B)** Evolution of the killing function (fKkill) and cytokine release function (fK_Cyt_) over time with two TCE administrations 7 (upper panel) or 21 (lower panel) days apart. The gray arrows represent the TCE dosing events (same dose).

The model was then used to explore the behavior of the different model entities under different scenarios (**Figure 3**). In the proposed PKPD model, it is assumed that IL6 is released only in presence of naive T-cells, and that the amount released is proportional to the quantity of naive T-cells available at the time of the second TCE administration. Accordingly, the model posits that as almost no naive T-cells are available 7 days after a first TCE administration, if a second dose were to be administered, no additional increments in activated T-cells would be observed, neither in IL6 release. Tumor cell killing would be maintained by the presence of desensitized T-cells (**Figure 3B**). On the other hand, 21 days after the first TCE administration, the PKPD model assumes that naive T-cells levels are close to their baseline values, which leads to IL6 levels similar to those observed following the initial TCE administration if a second TCE stimulation occurs on that day (**Figure 3B**).

**Figure 4:**
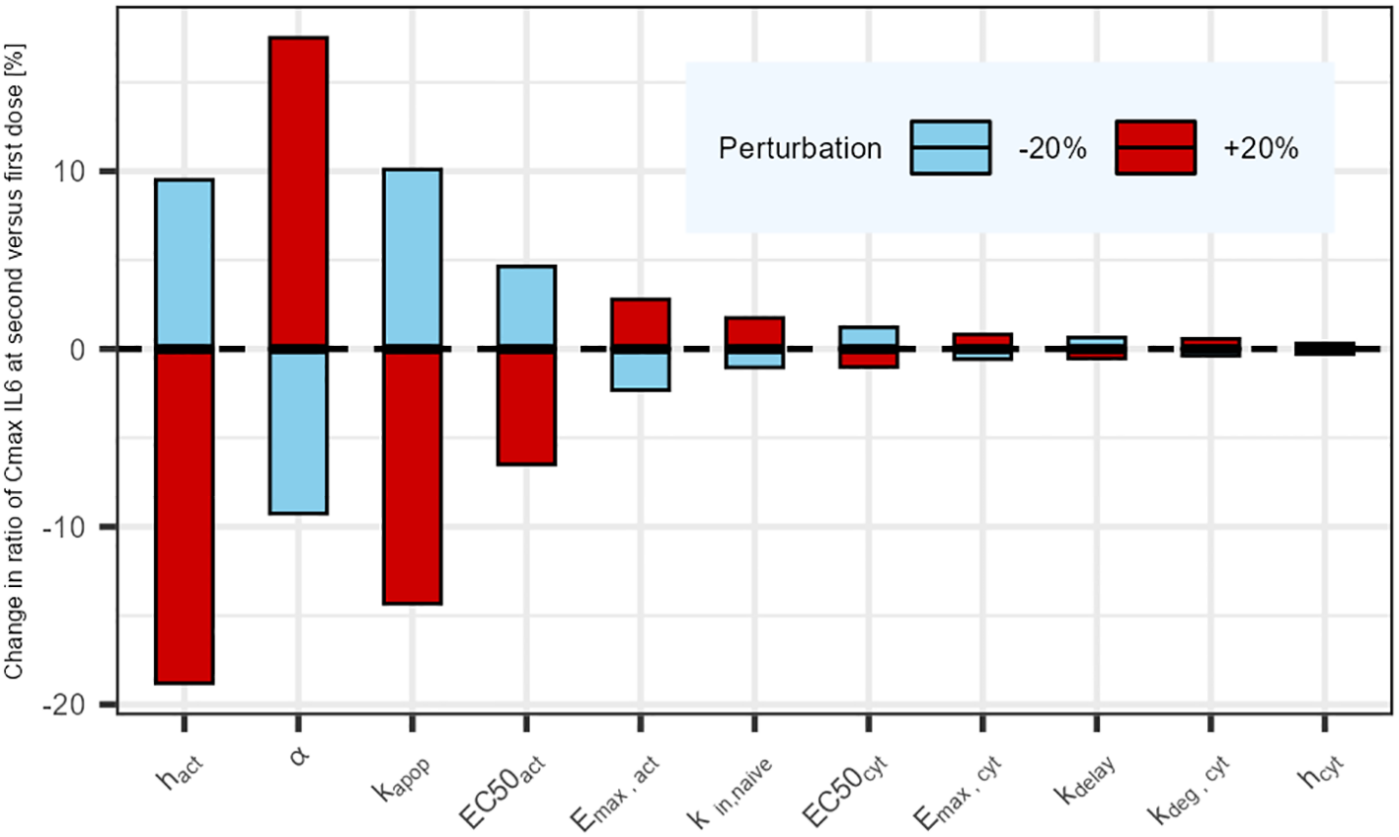
Parameter sensitivity analysis: Impact of a sequential 20% deviation on each parameter on the simulated ratio of IL6 maximum concentration at first administration versus second administration 21 days later. Parameters influencing tumor cell killing were omitted (not affecting cytokine release).

The results of the parameter sensitivity analysis for a dosing interval of 21 days are presented in **Figure 4**. The analysis revealed that the parameters with highest influence on IL6 release predictions are the rate constants associated with naive T-cells (k_in,naive_ and k_apo,naive_), as well as the parameters associated with the cytokine release function in relation to plasma concentration (α, Emax_Cyt_ and EC50_Cyt_). The same parameters were highlighted by the sensitivity analysis that was performed for the other tested dosing intervals. The alpha (α) parameter influences the dynamics of the cytokine release (eg, return to baseline after C_max_). Its value could be informed with a dataset capturing the dynamics of cytokines after C_max_.

## Discussion

TCEs have shown promising potential in cancer therapy, but their clinical development can be risky for patients due to their propensity of inducing cytokine release, a potentially life-threatening condition. Cytokine blockage agents like Tocilizumab are often used as a rescue method for high grades CRS. Conservative approaches like the *in vitro* minimum anticipated biological effect level (MABEL) approach (8) are also recommended to prevent high grades of CRS for the first-in-human (FIH) dose selection. Nevertheless, the *in vitro* MABEL derived FIH dose can be substantially lower than the therapeutic dose, leading to long dose escalation phases (12). Consequently, with this approach, many patients are treated at sub-therapeutic doses while some others are put at risk when approaching the Maximum Tolerated Dose (MTD). The final recommended dose and schedule for the drug label will be empirically decided based on all evidence gathered during the clinical development phase. The occurrence of severe CRS in the clinic can also be mitigated by using a SUD approach. Nevertheless, the determination of the optimal SUD dosing regimen remains a major challenge and is often the result of an empirical approach involving many patients. Hereby, a mathematical model capable of predicting cytokine release during the first two TCE administrations is presented. It brings quantitative insights on the dosing interval for the step-up dosing procedure that could be used for any CD3 TCE. The mechanistic PKPD model can be eligible for a Model-Informed Drug Development (MIDD) approach to prospectively design clinical studies (33). It can serve to inform dosing regimen to minimize cytokine release, potentially reducing the CRS risk for the patients and hopefully shortening the early phases of clinical development. It can also be used in a sequential approach to refine the next cohort dosing regimen based on emerging clinical data, similar to the methodology used for the Escalation With Overdose Control (EWOC) clinical trial design (34).

The presented mechanistic PKPD model was expanded from previously published models (17–19) to integrate newly available data (16). In the absence of PK data from this study, the PKPD model uses a simple one-compartment PK model to capture TCE concentration. This choice provides a straightforward framework that can be adjusted based on the specific properties of the studied molecule such as Target-Mediated-Drug-Disposition (TMDD) or FcRn recycling (35). The mechanistic PKPD model describes the main findings from Li et al. (decreased cytokine release upon second TCE administration) as a shift in the T-cell phenotype, and as opposed to the loss of tumor cells suggested in previous models (17, 18). The proposed mechanistic PKPD model incorporates three T-cell states as in Hosseini and colleagues (18), with the difference that upon dosing, the T-cells move to a desensitized state in which they can no longer release cytokines but retain their cytotoxic potential (as suggested by (16, 36)). Here, it is assumed that there is an initial limited pool of naive T-cells available for activation (and therefore available for cytokine release) which takes time to fully replenish and prevent cytokine release upon following TCE administrations. Upon first TCE stimulation, the pool of baseline naive T-cells transition to an activated T-cell status able to release cytokines. Those T-cells transit then to a status referred to as desensitized, by losing their ability to release cytokines. After this first TCE stimulation, it takes about 28 days to fully replenish the pool of baseline T-cells (in absence of any new stimulation), and retrieve the cytokine levels following the first TCE administration. In the meantime, any new TCE stimulation will lead to less cytokine release compared to the first TCE stimulation, as the number of naive T-cells is also lower. A shorter dosing interval, as opposed to a longer one, will result in reduced systemic release of cytokines. The model simulation behaves similarly to the clinical observations where a step-up dosing approach (a priming low dose is given followed by a higher dose within a short dosing interval) is taken. Cytokine levels are reduced after the second dose, despite it being higher than the first one (12, 37). With one dose level tested *in vivo* (0.5mg/kg), it was not possible to establish a relationship between drug exposure, T-cell activation and consequently cytokine release. Thus, the model assumes a complete transfer of the naive T-cells to activated T-cells at the tested dose. To allow for an increase in cytokine release, the release function (fK_cyt_) depends thus on both the amount of available T-cells and the drug concentration. The principle of an initial reservoir (pool) of biological entities to be emptied by drug exposure was first proposed by Movin-Osswald and Hammarlund-Udenaes (20) in a model referred to as the “pool model” (Appendix). In the pool model, the group assumed that an initial pool of prolactin (PRL) is depleted following drug exposure and takes time to replenish. The original pool model was repurposed to describe cytokine release (**Supplementary Figure S2**, Appendix) and its performance was compared to the presented PKPD model. Both models performed equally well (**Supplementary Figure S3**, Appendix). Nevertheless, the newly proposed PKPD model provides a new interpretation of the data and integrates new components considered essential for further development and use of the model, such as the tumor response that can be uncoupled from cytokine release upon second TCE administration, as suggested *in vitro* (16). T-cell proliferation is also included in the mechanistic PKPD model as it is an essential element to describe the individual patient’s response in the clinic (extent of tumor killing, delay in response…). An empirical approach driven by the E:T ratio was used to describe the proliferation effect, which is assumed to be driven by IL2 (IL2_effect_) but can in reality integrate indirectly other mechanisms. At high ratios (above 10), the proliferation effect is inhibited to avoid an infinite T-cell proliferation. Various processes like IL2 downregulation or regulatory T-cells may explain this inhibition. The choice of the E:T ratio was driven by *in vitro* knowledge since the *in vivo* E:T ratio was unknown. It must be noted that this may not reflect the *in vivo* and/or clinical conditions. With the proposed mechanistic PKPD model, T-cells count and status could be used as biomarkers to inform on individual dosing regimen, allowing for personalized dosing.

The presented model was developed with the goal of providing a theoretical framework for understanding cytokine release dynamics upon the first two TCE administrations and generating hypotheses, ultimately contributing to the optimization of TCE dosing regimens. The PKPD model suggests biological mechanisms that can be tested in preclinical or clinical settings. In particular, *in vivo* longitudinal studies of cytokine release, T-cell state and evolution, and tumor cell killing for different dosing regimens (dose levels, number of doses, dosing intervals) would better characterize the relationship between T-cell number/state and cytokine release. Consequently, this would allow to better inform the activation function of the T-cells (fKact), potentially removing the dependency on drug plasma concentration for cytokine release. The model was developed based on *in vitro* and *in vivo* data, and clinical data would be needed to confirm the model predictions. In particular, testing different dose levels would also enable the refinement of the relationship between cytokine release and TCE exposure after the first dose. Furthermore, due to the true complexity of the immune system and of the mechanisms involved in cytokine release, as well as the lack of available data, some simplifications were made in the model. For instance, the cellular source of cytokines can be diverse and the presented model considers the cytokine release from T-cells only. As suggested in (15, 22), monocytes or macrophages may become activated following T-cell activation triggered by TCE, potentially contributing to additional cytokine release. In addition, the parameters for T-cell kinetics may differ from reality. Specifically, the assumption of identical rates for activation and desensitization (k_delay_) may not accurately represent the underlying biological processes, and some parameters (k_in,naive_, k_apop_) were derived from human data while simulating cytokine release in mice. Moreover, the model could be updated with more data to link the cytokine release to the trimeric complex formation (TCE, T-cell, tumor cell), as in the model from Jiang et al. (17). This would better explain the variability observed in the patient population potentially due to the differences in tumor burden and T-cell levels.

## Conclusion

A new mechanistic PKPD model has been developed to describe IL6 cytokine levels associated with the first and second TCE doses with varying dosing intervals between the two administrations. In the proposed model, the decrease of cytokine release upon second TCE administration observed in Li et al. (16) is hypothesized to result from a transition of T-cells, and not from the loss of tumor cells. The shift takes the T-cells from a state capable of cytokine release to another state where they are not capable of such release. In particular, the model is able to link the second administration cytokine levels to the interval versus the first TCE administration. The hypotheses stated in the model are thought to be valid for any CD3-TCE and could thus be used for the drug development of multiple molecules. The model suggests refined pre-clinical experiments to further support drug development by providing more mechanistic insights on the TCE associated cytokine release. It also opens opportunities to leverage PKPD modeling during the clinical development, potentially increasing the likelihood of success of developing new TCE drugs, by moving quicker in optimized drug dosing schemes, maximizing TCE associated patient’s benefits while minimizing the risk of CRS.

## Data Availability

The original contributions presented in the study are included in the article/**Supplementary Material**. Further inquiries can be directed to the corresponding author.
