# Supplementary material for: Mechanistic PKPD modeling to describe cytokine release associated with CD3 T-cell engager therapies
Source: Front Immunol. 2025 Jan 17;15:1463915. doi: 10.3389/fimmu.2024.1463915 (PMC11782561; doi:10.3389/fimmu.2024.1463915)
Supplement: Supplementary file 1 [file DataSheet1.docx]

**Mechanistic PKPD modeling to describe cytokine release associated with CD3 T-cell engagers therapies**

**Supplementary material:**

Mechanistic PKPD model

Table S.I: IL6 plasma levels from the mice experiment, digitized from Li et al. 2019 [1]

| **Dosing interval** | **IL6 levels [pg/mL]** | **Dose number** |
| --- | --- | --- |
| D0 | 100.00 | First |
| D1 | 10.00 | Second |
| D7 | 11.25 | Second |
| D14 | 16.60 | Second |
| D21 | 46.10 | Second |
| D28 | 87.70 | Second |

TNF𝛼

The structure of the model also allows to describe the time course of other cytokines, like TNF𝛼, which show similar dynamics as IL6 following the administration of TCE at different dosing intervals. The model simulations are displayed in **Fig.S1** and show that general tendency is well captured by the model. The peak levels, replenishment dynamics and baseline concentrations of TNF𝛼 differ from IL6, requiring updates of the parameters E_max,Cyt_ and h_act_ (steep) and baseline for TNF𝛼. The updated parameters are displayed in **Table S.II**.


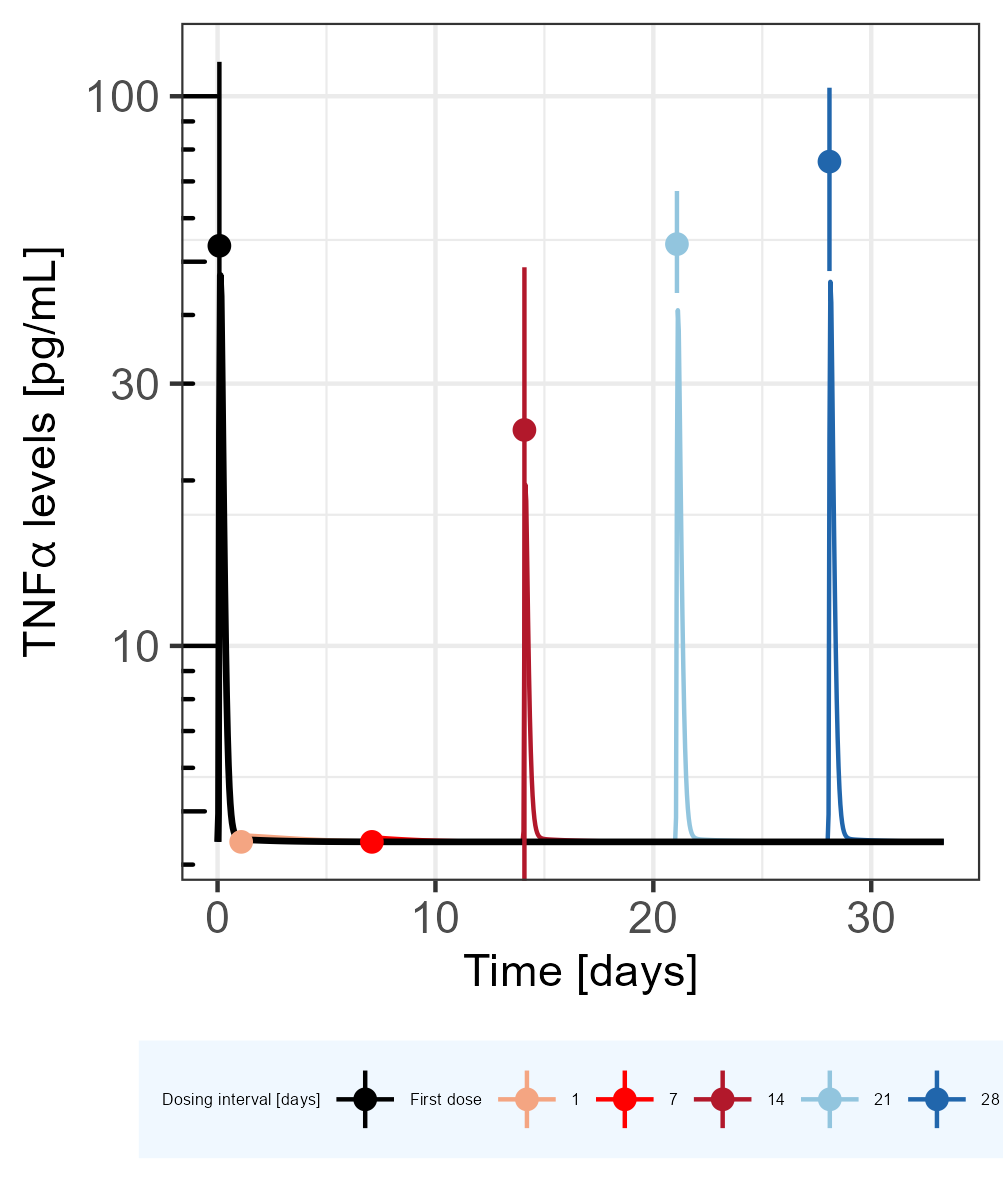


**Figure S1: Model predicted TNF𝛼 time course (solid lines) following two consecutive TCE administrations at different dosing intervals (administrations at day 0 and day 1; day 0 and day 7; day 0 and day 14; day 0 and day 21 or day 0 and day 28). Dots represent the digitized experimental data from Li et al. (2019) along with their standard error of mean (SEM).**

Table S.II: Updated model parameters for TNF𝛼

| **Parameters** | E_max,cyt_ | h_act_ | TNF𝛼, baseline |
| --- | --- | --- | --- |
| **Updated value** | 0.00096 | 3.5 | 4.4pg/mL |

Pool model

The model from Movin-Osswald and Hammarlund-Udenaes [2],  referred to as the “Pool model”, describes the effect of Remoxipride on prolactin release (PRL) via an indirect response model. Hereby, the original model was repurposed to describe the time course of cytokine levels after two first consecutive doses of TCE at different dosing intervals. This model implies that there is a limited pool of cytokines (eg. contained in T-cells or other immune cells) that are available for release upon TCE administration. Upon the first administration, the pool of cytokines is depleted and needs time to replenish to baseline levels. The structure of the pool model is described in **Fig.S2**.


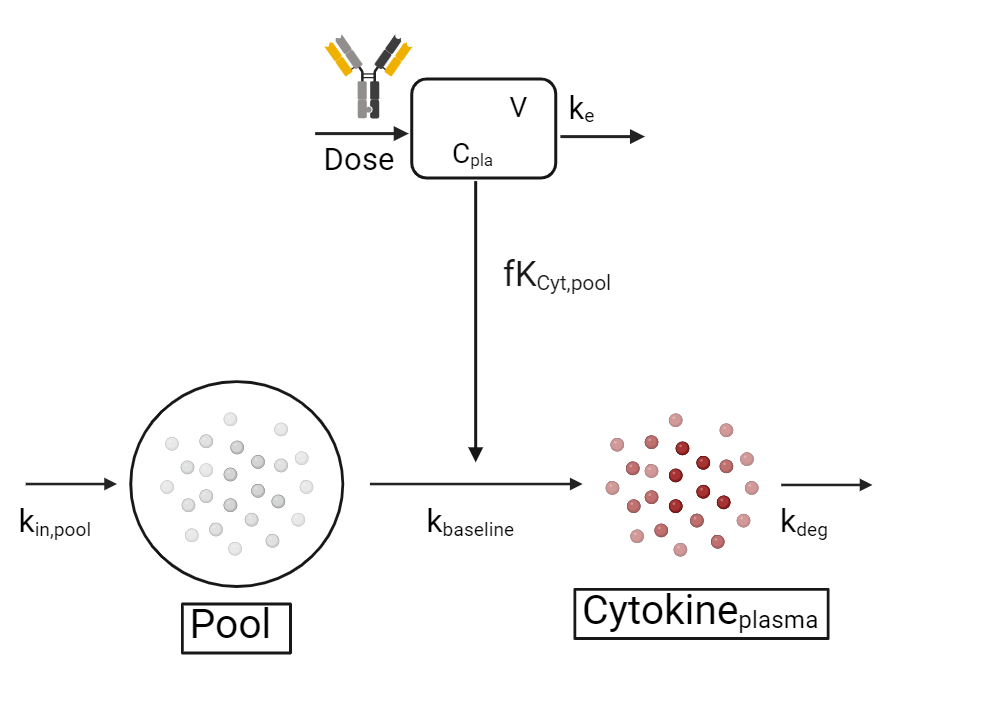


**Figure S2: Graphical representation of the pool model repurposed to describe cytokine release in plasma following TCE administration. Figure created with Biorender.com.**

The repurposed pool model parameters were estimated from the *in vivo* cytokine data from Li et al. [1] using the naive pool approach in the Monolix software [3]. The final parameters are displayed in **Table S.III**. The linearization method was used to compute the standard errors as no population parameters were introduced and a proportional error model (with parameter b) was used.  Similarly to the mechanistic PKPD model, the TCE plasma concentration (C_pla_) was assumed to follow a 1-compartment PK (Eq.S1), and the PK parameters (volume of distribution V and elimination rate constant k_e_) and the degradation rate of cytokines (k_deg_) were fixed ([4], [5]). The pool of cytokines is synthesized at rate constant k_in,pool_ and releases cytokine into plasma at rate constant k_baseline_ (Eq.S2). Upon TCE administration, the cytokine release will be influenced by the drug concentration through fK_Cyt,pool_.  The equations for the pool model are as follows:

$$\frac{dC_{pla}}{dt}= - \frac{CL}{V}\times C_{pla} [Eq.S1]$$

$$C_{pla}\left( t=0 \right)=0$$

$$\frac{dC_{pool}}{dt}=k_{in,pool}-k_{baseline}\times fK_{Cyt,pool}\times C_{pool} [Eq.S2]$$

$$C_{pool}\left( t=0 \right)= \frac{k_{in,pool}}{k_{baseline}}$$

$$\frac{dC_{cytokine}}{dt}=k_{baseline}\times fK_{Cyt,pool}\times C_{pool}-k_{deg}\times C_{cytokine} [Eq.S3]$$

$$C_{cytokine}\left( t=0 \right)= \frac{k_{in,pool}}{k_{deg}}$$

The function for cytokine release (fK_Cyt,pool_) was modeled by an Emax model, as shown in Eq. S4:

$$fK_{Cyt,pool}=1+E_{max,cyt}\times\left( \frac{C_{pla}}{C_{pla}+EC50} \right) [Eq.4]$$

The estimated parameters and their relative standard errors (%) are displayed in **Table S.II:**

Table S.III: Parameters of the pool model, repurposed to describe cytokine release, along with their values and relative standard errors.

| **E_max,cyt_** | **EC50** | **k_in,cyt_** | **k_baseline_** | **k_deg_** | **b** |
| --- | --- | --- | --- | --- | --- |
| 95.5  (35.7%) | 1.2  (33.6%) | 3.92  (4.90%) | 0.02  (8.09%) | 0.41  (fixed) | 0.06  (28.9%) |

The pool model was able to describe the *in vivo* data from Li et al. In particular, it was able to describe a low level of cytokine release when the dosing interval is too short (one to seven days). The pool model simulations for different dosing intervals are displayed in **Fig.S3** and compared to the mechanistic PKPD model simulations.


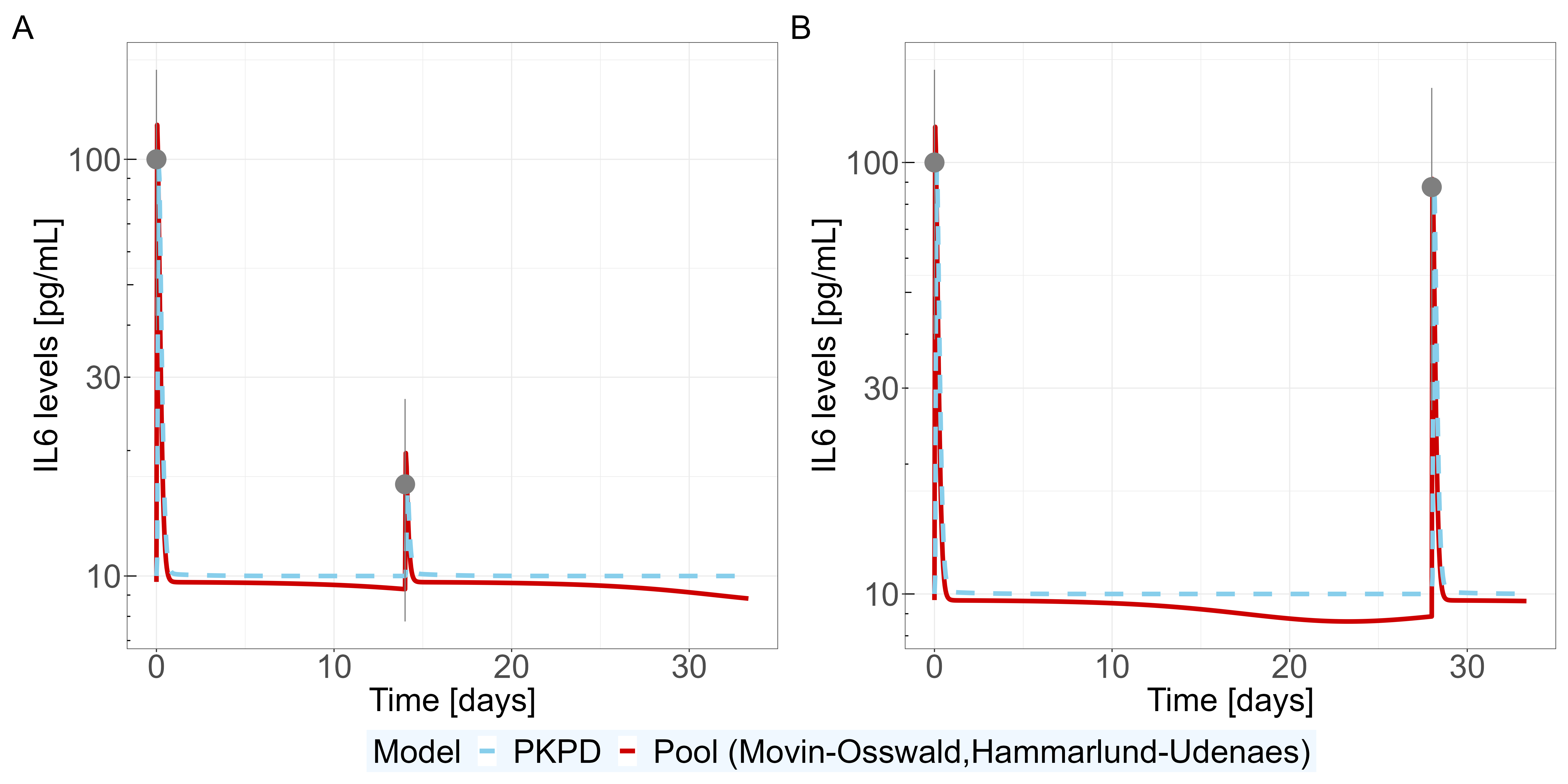


**Figure S3: Comparison of simulations obtained with the PKPD model and the Pool model for a dosing interval of 14 days (A) or 28 days (B). The grey dots represent the mean cytokine levels measured in mice [1] along with the standard error of the mean (SEM).**

**Bibliography (Supplementary material)**

1. Li J, Piskol R, Ybarra R, Chen YJ, Li J, Slaga D, et al. CD3 bispecific antibody-induced cytokine release is dispensable for cytotoxic T cell activity. Sci Transl Med. 2019;11(508). doi: 10.1126/scitranslmed.aax8861.

2. Movin-Osswald G, Hammarlund-Udenaes M. Prolactin release after remoxipride by an integrated pharmacokinetic-pharmacodynamic model with intra- and interindividual aspects. J Pharmacol Exp Ther. 1995;274(2):921-7.

3. Monolix version 2021R2: Lixoft SAS. R2 ed. Anthony, France2021.

4. Yu S, Zhang J, Yan Y, Yao X, Fang L, Xiong H, et al. A novel asymmetrical anti-HER2/CD3 bispecific antibody exhibits potent cytotoxicity for HER2-positive tumor cells. J Exp Clin Cancer Res. 2019;38(1):355. doi: 10.1186/s13046-019-1354-1.

5. Frances N, Bacac M, Bray-French K, Christen F, Hinton H, Husar E, et al. Novel in Vivo and in Vitro Pharmacokinetic/Pharmacodynamic-Based Human Starting Dose Selection for Glofitamab. J Pharm Sci. 2022;111(4):1208-18. doi: 10.1016/j.xphs.2021.12.019.
